# Association of State Social and Environmental Factors With Rates of Self-injury Mortality and Suicide in the United States

**DOI:** 10.1001/jamanetworkopen.2021.46591

**Published:** 2022-02-09

**Authors:** Ian R. H. Rockett, Haomiao Jia, Bina Ali, Aniruddha Banerjee, Hilary S. Connery, Kurt B. Nolte, Ted Miller, Franklin M. M. White, Bernard D. DiGregorio, G. Luke Larkin, Steven Stack, Kairi Kõlves, R. Kathryn McHugh, Vijay O. Lulla, Jeralynn Cossman, Diego De Leo, Brian Hendricks, Paul S. Nestadt, James H. Berry, Gail D’Onofrio, Eric D. Caine

**Affiliations:** 1 Department of Epidemiology and Biostatistics, School of Public Health, West Virginia University, Morgantown; 2 Department of Psychiatry, University of Rochester Medical Center, Rochester, New York; 3 Pacific Institute for Research and Evaluation, Calverton, Maryland; 4 Department of Geography, Indiana University–Purdue University at Indianapolis; 5 McLean Hospital, Belmont, Massachusetts; 6 Department of Psychiatry, Harvard Medical School, Boston, Massachusetts; 7 Departments of Pathology and Radiology, University of New Mexico School of Medicine, Albuquerque; 8 Departments of Pathology and Radiology, University of New Mexico School of Medicine, Albuquerque; 9 Centre for Population Health Research, Curtin University, Perth, Australia; 10 Department of Community Health and Epidemiology, Dalhousie University, Halifax, Canada; 11 Department of Sociology and Anthropology, West Virginia University, Morgantown; 12 Northeast Ohio Medical University, Rootstown; 13 Departments of Criminal Justice and Psychiatry and Behavioral Neurosciences, Wayne State University, Detroit, Michigan; 14 Australian Institute for Suicide Research and Prevention, Mount Gravatt, Australia; 15 WHO Collaborating Centre for Research and Training in Suicide Prevention, Griffith University, Mount Gravatt, Australia; 16 College for Health, Community and Policy, University of Texas, San Antonio; 17 Slovene Centre for Suicide Research and Department of Psychology, University of Primorska, Koper, Slovenia; 18 Department of Psychiatry and Behavioral Sciences, Johns Hopkins School of Medicine, Baltimore, Maryland; 19 Department of Behavioral Medicine and Psychiatry, West Virginia University, Morgantown; 20 Department of Emergency Medicine, Yale School of Medicine, New Haven, Connecticut; 21 Department of Biostatistics, Mailman School of Public Health and School of Nursing, Columbia University, New York, New York

## Abstract

**Importance:**

Self-injury mortality (SIM) combines suicides and the preponderance of drug misuse–related overdose fatalities. Identifying social and environmental factors associated with SIM and suicide may inform etiologic understanding and intervention design.

**Objective:**

To identify factors associated with interstate SIM and suicide rate variation and to assess potential for differential suicide misclassification.

**Design, Setting, and Participants:**

This cross-sectional study used a partial panel time series with underlying cause-of-death data from 50 US states and the District of Columbia for 1999-2000, 2007-2008, 2013-2014 and 2018-2019. Applying data from the Centers for Disease Control and Prevention, SIM includes all suicides and the preponderance of unintentional and undetermined drug intoxication deaths, reflecting self-harm behaviors. Data were analyzed from February to June 2021.

**Exposures:**

Exposures included inequity, isolation, demographic characteristics, injury mechanism, health care access, and medicolegal death investigation system type.

**Main Outcomes and Measures:**

The main outcome, SIM, was assessed using unstandardized regression coefficients of interstate variation associations, identified by the least absolute shrinkage and selection operator; ratios of crude SIM to suicide rates per 100 000 population were assessed for potential differential suicide misclassification.

**Results:**

A total of 101 325 SIMs were identified, including 74 506 (73.5%) among males and 26 819 (26.5%) among females. SIM to suicide rate ratios trended upwards, with an accelerating increase in overdose fatalities classified as unintentional or undetermined (SIM to suicide rate ratio, 1999-2000: 1.39; 95% CI, 1.38-1.41; 2018-2019: 2.12; 95% CI, 2.11-2.14). Eight states recorded a SIM to suicide rate ratio less than 1.50 in 2018-2019 vs 39 states in 1999-2000. Northeastern states concentrated in the highest category (range, 2.10-6.00); only the West remained unrepresented. Least absolute shrinkage and selection operator identified 8 factors associated with the SIM rate in 2018-2019: centralized medical examiner system (β = 4.362), labor underutilization rate (β = 0.728), manufacturing employment (β = −0.056), homelessness rate (β = −0.125), percentage nonreligious (β = 0.041), non-Hispanic White race and ethnicity (β = 0.087), prescribed opioids for 30 days or more (β = 0.117), and percentage without health insurance (β = −0.013) and 5 factors associated with the suicide rate: percentage male (β = 1.046), military veteran (β = 0.747), rural (β = 0.031), firearm ownership (β = 0.030), and pain reliever misuse (β = 1.131).

**Conclusions and Relevance:**

These findings suggest that SIM rates were associated with modifiable, upstream factors. Although embedded in SIM, suicide unexpectedly deviated in proposed social and environmental determinants. Heterogeneity in medicolegal death investigation processes and data assurance needs further characterization, with the goal of providing the highest-quality reports for developing and tracking public health policies and practices.

## Key Points

**Question:**

Do state rates of SIM and suicide vary with social factors and administrative reporting systems?

**Findings:**

This cross-sectional study including 101 325 deaths found that social and administrative factors were associated with interstate variation in SIM and suicide rates, and adjusted SIM rates were higher in medical examiner states with centralized authority, implying better detection of fatal drug poisonings.

**Meaning:**

These findings suggest that SIM and suicide and related interventions need to address broad social factors from inequity to health care access to injury means.

## Teaser

This cross-sectional study assesses factors associated with interstate variations in self-injury mortality and suicide rates.

## Introduction

The United States has endured an evolving crisis of self-injury mortality (SIM) during the opening decades of the 21st century due to suicide and drug misuse.^1^ SIM augments registered suicides with most opioid or other drug overdose fatalities caused by individuals’ instrumental actions on the day of death—ultimately with the goal of improving injury surveillance, enhancing etiologic understanding, and establishing the foundation for effective, scalable programs of prevention and clinical intervention.^2,3^ We have applied the broader construct of SIM to characterize drug fatalities arising from persons’ instrumental behaviors, given well-documented uncertainty of where to draw demarcating lines between deaths arising from an intention to die (ie, suicides), intentional behaviors that lead to fatalities (ie, drug-related deaths now called *accidents*, later relabeled *unintentional* by the Centers for Disease Control and Prevention) as diagnosed by medical examiners or ruled by coroners , as well as truly accidental or unintentional injuries that occur without preceding probability-altering risk factors.

The aims of this study were to identify administrative and contextual factors associated with both state-level SIM and suicide rates, and to explore the differential potential of regions and states to undercount suicides owing to misclassification of drug-related fatalities. Medical examiners and coroners either have had insufficient investigative resources or lacked definitive evidence of intent to cause one’s death (eg, an authenticated suicide note),^4^ typically leading to a misclassified manner-of-death determination of *accident* or *undetermined*.^5^ Moreover, fatal drug misuse most often arises from intentional behaviors, such as patterns of self-harm involving motivated acquisition of drugs, recurring self-administration in the context of substance use disorder, and ultimately, death.^6^ Even when intent-to-die was not present on the day of death, these fatalities were neither an accident nor their antecedents unintentional.

This study addresses a third concern, also noted by others.^7^ While there are many apparent, shared individual psychological risk factors associated with both suicide and fatal nonsuicidal drug ingestions,^8,9^ there may be contextual factors common to SIM and suicide that are potentially amenable to broader public health-related interventions. We have sought to identify state-level factors associated with variation in SIM and suicide rates from a constellation of variables representing inequity and economic resources, social isolation, demographics, injury mechanism, and health care access.

Factored into our contextual analysis is an administrative environmental element, the heterogeneous nature of medicolegal death investigations,^10,11^ a variable seldom incorporated in US suicide studies,^4,5,12,13^ and never in a SIM study, to our knowledge. Heterogeneity could affect the accuracy of reported rates of SIM, as well as suicide. We used type of medicolegal death investigation system as a proxy for variable investigation quality.

**Figure 1.**
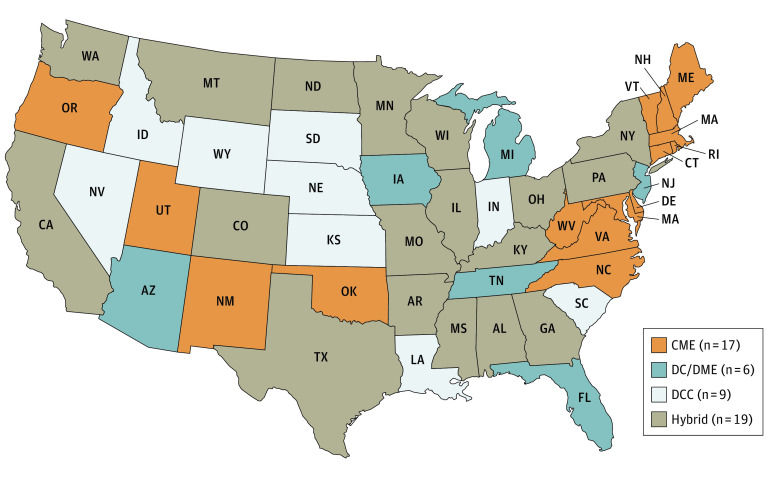
Type of Medicolegal Death Investigation System by State, 2018 CME indicates centralized medical examiner; DC/DME, decentralized county or district medical examiner; DCC, decentralized county or district coroner; and hybrid, medical examiner and coroner hybrid.

The US has a patchwork of medicolegal death investigative systems, principally divided between medical examiners and coroners, with some states exhibiting a combination of both types (Figure 1). These systems bring different skills, experience, and resources to the conduct of their investigations.^10^ Medical examiners are typically appointed physicians who have residency or fellowship training and board certification in pathology and forensic pathology. Medical examiner offices have jurisdictions that can encompass an entire state, a region of a state, a county, or a city. Most medical examiner offices provide comprehensive death investigation services that often include other specialists, such as field investigators, toxicologists, and forensic anthropologists. By contrast, coroners are generally lay individuals who are elected to their posts and rarely physicians. Coroner offices function at a county level; sometimes they employ trained field investigators, although they usually contract with medical examiner offices or forensic pathologists for autopsy and other specialty services on an as-needed basis. States having a mix of systems often have medical examiners in population-dense urban areas and coroners in more rural counties. In general, offices that serve larger jurisdictions have an economy of scale that supports more advanced technology (eg, computed tomography) and laboratory services. Centralized state medical examiner systems foster uniformity of procedures and more robust population-based data. Suicide measurement is conservative,^14^ and determining suicide by opioid and other drug self-intoxication is especially difficult for medical examiners and coroners to ascertain.^4,15,16,17^ The validity of manner-of-death certification by coroners has been shown to be significantly reduced when medication or drugs of abuse are involved.^18^ While coroner offices typically manifest the greatest operational deficiencies, all medicolegal death investigation offices tend to be underresourced, especially since the Great Recession, and even more so with the explosion of opioid deaths during recent years.^19,20,21^

Preceding the contextual analysis of interstate SIM and suicide rates in 2018-2019, we graphed and mapped changes in SIM to suicide rate ratios (RRs) in the US since the turn of the century. This was to assess the potential for differential suicide misclassification by state and major region amidst the opioid overdose epidemic.^1^

## Methods

This cross-sectional study was a secondary analysis of an aggregated, state-level, publicly accessible mortality data set; therefore, it was deemed exempt from an ethical evaluation and informed consent by the institutional review board of West Virginia University. This study is reported according to the Strengthening the Reporting of Observational Studies in Epidemiology (STROBE) reporting guideline.

### Data Sources and SIM Measurement

We used deidentified manner and underlying cause-of-death data for the 50 US states and the District of Columbia at 4 observation points: 1999-2000, 2007-2008, 2013-2014, and 2018-2019; rationale for these points has been previously documented.^1^ Our source was CDC’s Wide-ranging Online Data for Epidemiologic Research (WONDER) database.^22^ Deaths had been precoded in accordance with the *International Statistical Classification of Diseases and Related Health Problems, Tenth Revision* (*ICD-10*).^23^ Elaborated in our complementary study,^1^ SIM was operationalized as the sum of all suicides (*ICD-10* codes UO3, X60-X84, and Y 87.0) by any injury mechanism or method, irrespective of decedent age, and 80% of accidental (*unintentional* using CDC nomenclature) opioid, alcohol, and other drug intoxication deaths (*ICD-10* codes X40-X45) and 90% of corresponding undetermined intent drug intoxication deaths (*ICD-10* codes Y10-Y15) among persons ages 15 years and older. The proportions for the undetermined and unintentional drug deaths were applied directly to the aggregated data to estimate the active behavior component of SIM.^1,2,3^ We assumed these deaths were deaths from drug self-intoxication,^6^ as potentially verifiable by singular or cumulative evidence of pharmacy or physician shopping in a prescription drug monitoring database, high concentration of drugs in the stomach or small intestine of the corpse detected through autopsy, illicit drug positivity in toxicologic testing, and documentation of drug paraphernalia at the death scene.^3^ This active behavior component reflects common patterned, premorbid behaviors associated with increased risk for premature death.^2^ The proportion was higher for the undetermined deaths because those medicolegal assignments allow for the possibility that a given death was due to completed suicide, accident, or homicide. However, homicides comprised only 0.2% of drug intoxication deaths vs 10% for suicide and 80% for unintentional injury during the period 2009 to 2018,^22^ making the misclassified homicide share of undetermined drug deaths negligible. The age 15 years cutoff for inclusion in the active behavior component of SIM assumed relative rarity of purposive self-harm behaviors among children and younger adolescents.^24^

We computed 2-year annualized counts and crude mortality rates to stabilize the data. In mapping the SIM to suicide RRs by state and period, the quartile distribution of values in 2018-2019 was used to determine the comparative categories. We identified the IQR to approximately span the values of 1.5 and 2.1, since it contained 50% of the RRs. Consequently, we used the equal sized intervals method to subdivide the RRs into 4 groups for optimized representation.

### Multivariable Modeling

In modeling potential state-level, contextual factors associated with variation in respective annualized crude SIM and suicide rates for multivariable regression analyses, we included socioeconomic indicators previously associated with suicide and fatal overdose risk,^7,25,26,27,28,29,30^ and other variables representing population demographics, injury mechanism, social isolation, health care access, and an administrative variable, medicolegal death investigation system type.

Nine variables represented inequity and economic resources: median household income, percentage of the population 25 years and older with less than a bachelor’s degree, economic dependency ratio (population aged 0-14 years and 65 years and older divided by the population aged 15-64 years), percentage of workforce employed in manufacturing (anticipated negative association), percentage of employed persons who are trade union members (anticipated negative association), poverty rate, labor underutilization rate, consumer bankruptcy rate, and housing foreclosure rate. Nine variables represented social isolation and separation: percentage of single-person households, percentage of residentially unstable persons, percentage of not-married persons, homelessness rate, incarceration rate, homicide rate, percentage of ballots counted for eligible voters (anticipated negative association), percentage of persons who are nonreligious, and percentage of persons without broadband service.

Representing demographic factors were percentage of persons who are male, percentage of persons who are non-Hispanic White, percentage of persons who are civilian veteran (ex-military), rurality, and major geographic region (ie, Northeast, South, Midwest, and West). Non-Hispanic White race and ethnicity was identified through application of the Hispanic origin and race variables in our mortality data source. Rural residence was determined by the 2010 US census population enumeration (eAppendix in the Supplement). Injury mechanism was represented by alcohol consumption per capita, percentage of the population owning a firearm, percentage of persons prescribed opioids for 30 days or more, percentage of persons using an illicit drug other than marijuana in the past month, and percentage of persons misusing a pain reliever in the past year. Health care access was represented by percentages of adults without a usual place of medical care, percentage of persons with unmet mental health treatment need, percentage of persons with no health insurance, and percentage of persons with an unfavorable disability claim ruling.

Types of medicolegal death investigation system were distinguished as centralized (state) medical examiner, decentralized county or district medical examiner, medical examiner and coroner hybrid, or decentralized county or district coroner.^10,11^ We compared states by selected system type with all others. Comprising multiple categories, both system type and major geographic region were represented as dummy variables.

### Statistical Analysis

Stepwise-based model selection methods, including forward selection and backward elimination, were inappropriate for this study owing to the small number of observations (51 observations), large number of possible highly collinear independent variables examined (33 variables), and potential influence of outliers. Consequently, we applied least absolute shrinkage and selection operator (LASSO) regression to identify the possible factors associated with the crude SIM rates and crude suicide death rates.^31^ The LASSO procedure can effectively identify important factors without overfitting by modifying coefficient estimation and reducing some unimportant variables to zero. LASSO performs model selection with improved identification, accuracy, and interpretability. To assess the performance of the selected model, modified by data availability, we used the social indicators proximal to 2016-2017 SIM and suicide data as the training data set, corresponding proximal 2018-2019 data as the testing data set, and the average square error of the testing data as the performance criterion. We concluded the selected model performed well if the average square error for the testing data was not much larger than that for the training data. Definitions and data sources for the variables are documented in the eAppendix in the Supplement.

Analysis was conducted using SAS statistical software version 9.4 (SAS Institute). Data were analyzed from February to June 2021.

## Results

### Visualization and Change in SIM to Suicide RR

**Figure 2.**
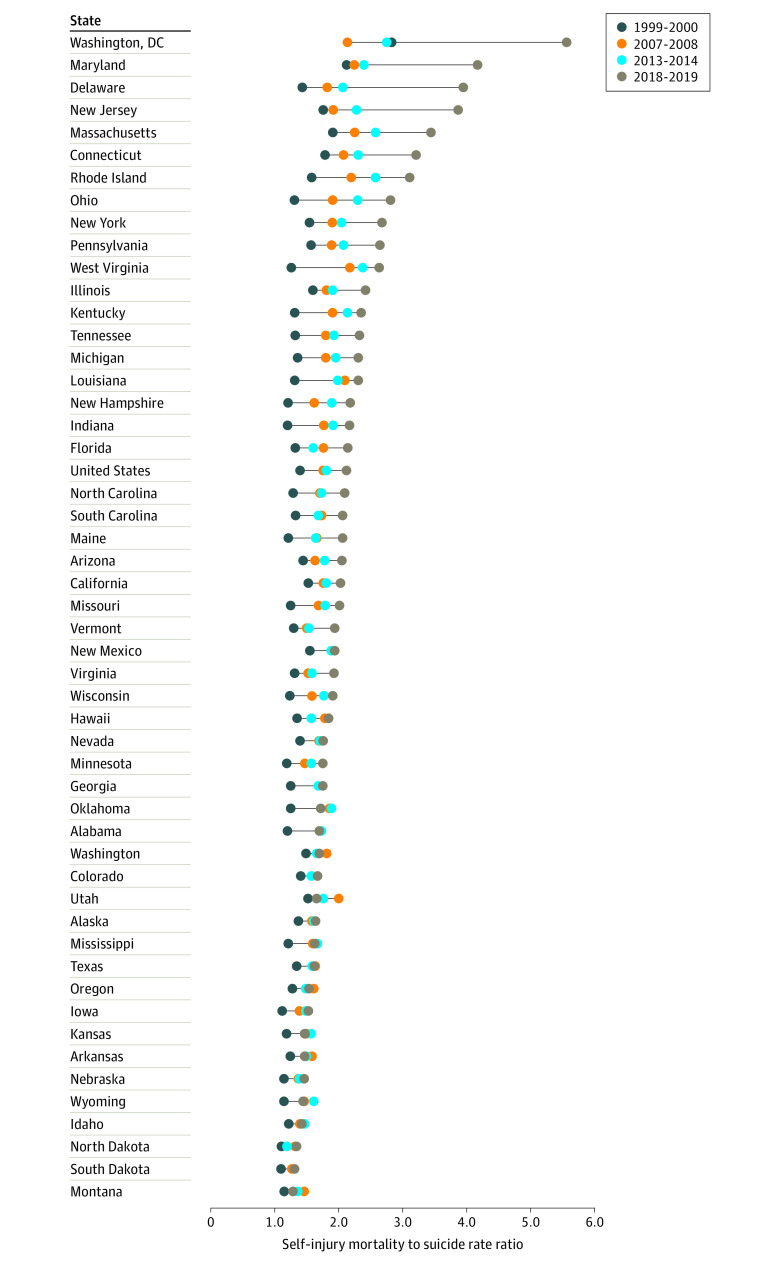
Graphed Annualized Crude Self-injury Mortality to Suicide Rate Ratios by State and Period

**Figure 3.**
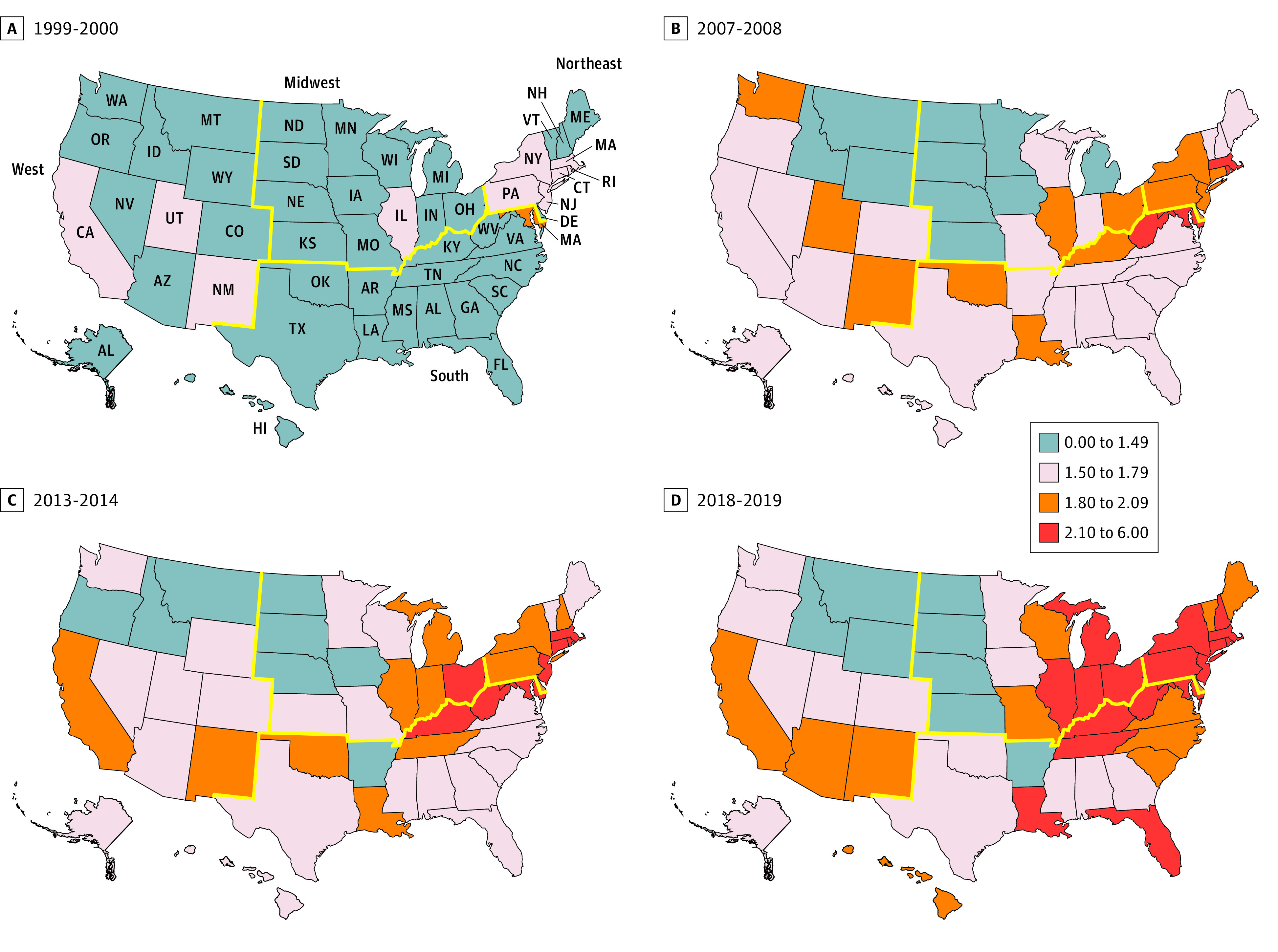
Mapped Annualized Crude Self-injury Mortality to Suicide Rate Ratios by State and Period Yellow lines indicate borders of geographic regions.

A total of 101 325 SIMs were identified, including 74 506 (73.5%) among males and 26 819 (26.5%) among females. Nationally, SIM to suicide RRs trended upwards over the observation period (Figure 2), reflecting the relatively higher rate of increase among registered overdose fatalities whose manner of death was classified as unintentional or undetermined (SIM to suicide RR, 1999-2000: 1.39; 95% CI, 1.38-1.41; 2018-2019: 2.12; 95% CI, 2.11-2.14). Mapping the SIM to suicide RRs provided a regional perspective (Figure 3). In 1999-2000, 39 states posted an RR less than 1.50, with all 4 major regions represented. This number had declined to 8 by 2018-2019, and only the West remained unrepresented in the highest category (range, 2.10-6.00). SIM to suicide RR gaps were narrowest in Western mountain states, adjacent Midwestern states, and Arkansas, and widest in a cluster of states that crossed Northeastern, Midwestern, and Southern boundaries and in 2 spatial outliers in the South, in Florida and Louisiana.

### Contextual Analysis of SIM and Suicide Rates, 2018-2019

Omitting type of medicolegal death investigation system, a modified LASSO model identified 9 factors associated with the SIM rate in 2018-2019—representing all 5 categories of source variables (Table 1). The SIM rate varied positively with the labor underutilization rate (β = 0.929) and negatively with percentage employed in manufacturing (β = −0.291) (inequity and economic resource variables). The SIM rate varied positively with percentage of population not married (β = 0.178) and percentage of population nonreligious (β = 0.131) and negatively with the homelessness rate (β = −0.270) (social isolation variables). Furthermore, SIM rate varied positively with percentage of population who were non-Hispanic White (β = 0.147) and was lower in the Midwest region (β = −1.029) than other regions combined (demographic variables). SIM rate varied positively with percentage of population prescribed opioids for 30 days (β = 0.183) (injury mechanism variable) and percentage of population without health insurance (β = −0.158) (health care access variable). The suicide rate varied positively with percentage of population that was male (β = 1.046), veteran (β = 0.747), and with rural residency status (β = 0.031) (demographic variables) and percentage firearm ownership (β = 0.030) and pain reliever misuse in the past year (β = 1.131) (injury mechanism variable).

**Table 1.** Models for US State-Based SIM and Suicide Rates Using Least Absolute Shrinkage and Selection Operator, Excluding Death Investigation System Type, 2018-2019

Parameter	SIM		Suicide	
	β	Standardized β^a^	β	Standardized β^a^
Intercept	1.168	0	−48.579	0
Inequity and economic resources				
Labor underutilization rate	0.929	0.191	NA	NA
% Manufacturing employment	–0.291	–0.132	NA	NA
Social isolation and separation				
% Not married	0.178	0.082	NA	NA
Homelessness rate	–0.270	–0.138	NA	NA
% Nonreligious	0.131	0.162	NA	NA
Demographics				
% Male	NA	NA	1.046	0.189
% Non-Hispanic White	0.147	0.295	NA	NA
% Military veteran	NA	NA	0.747	0.257
Midwest	–1.029	–0.055	NA	NA
% Rural	NA	NA	0.031	0.133
Injury mechanism				
% Firearm ownership	NA	NA	0.030	0.086
Opioids ≥30 d	0.183	0.243	NA	NA
% Pain reliever misuse	NA	NA	1.131	0.101
% Uninsured	–0.158	–0.060	NA	NA
CV PRESS	1816.533		222.329	
ASE				
Train	51.580		5.944	
Test	52.347		11.096	

Abbreviations: ASE, average square error; CV PRESS, cross-validation predicted residual sum of squares (obtained by summing the squares of the residuals when each of these submodels is scored on the data omitted in fitting the submodel); NA, not applicable; SIM, self-injury mortality.

^a^ A standardized regression coefficient was computed by dividing a parameter estimate by the ratio of the sample SD of the dependent variable to the sample SD of the regressor.

Adjusting for substantive factors with the inclusion of medicolegal system type in the model, central medical examiner states were associated with an excess SIM rate of 4.36 deaths per 100 000 population in 2018-2019 relative to remaining states (β = 4.362) (Table 2). While no new substantive factors emerged, 2 of 9 previously identified factors were eliminated: Midwest region and percentage of population not married. Labor underutilization rate (β = 0.728), manufacturing employment (β = −0.056), homelessness rate (β = −0.125), percentage nonreligious (β = 0.041), non-Hispanic White race and ethnicity (β = 0.087), prescribed opioids for 30 days or more (β = 0.117), and percentage without health insurance (β = −0.013) remained significantly associated. However, the 5 factors associated with the suicide rate, identified under the initial model, remained intact with the model substitution. Although SIM and suicide rates had none in common, some of their associated factors shared the broad categorical rubrics of demographic and injury mechanism variables.

**Table 2.** Final Model for State-Based, SIM and Suicide Rates Using Least Absolute Shrinkage and Selection Operator, United States, 2018-2019

Parameter	SIM		Suicide	
	β	Standardized β^a^	β	Standardized β^a^
Intercept	15.665	0	–48.579	0
Centralized medical examiner	4.362	0.259	NA	NA
Inequity and economic resources				
Labor underutilization rate	0.728	0.149	NA	NA
% Employed in manufacturing	–0.056	–0.025	NA	NA
Social isolation and separation				
Homelessness rate	–0.125	–0.064	NA	NA
% Nonreligious	0.041	0.05	NA	NA
Demographics				
% Male	NA	NA	1.046	0.189
% Non-Hispanic White	0.087	0.175	NA	NA
% Military veteran	NA	NA	0.747	0.257
% Rural	NA	NA	0.031	0.133
Injury mechanism				
% Firearm ownership	NA	NA	0.03	0.086
Opioids ≥30 d	0.117	0.156	NA	NA
% Pain reliever misuse	NA	NA	1.131	0.101
Uninsured (%)	–0.013	–0.005	NA	NA
CV PRESS	1431.289		222.329	
ASE				
Train	36.735		5.944	
Test	48.876		11.096	

Abbreviations: ASE, average square error; CV PRESS, cross-validation predicted residual sum of squares (obtained by summing the squares of the residuals when each of these submodels is scored on the data omitted in fitting the submodel); NA, not applicable; SIM, self-injury mortality.

^a^ A standardized regression coefficient is computed by dividing a parameter estimate by the ratio of the sample standard deviation of the dependent variable to the sample standard deviation of the regressor.

## Discussion

The findings of this cross-sectional study suggest that SIM rates were associated with modifiable, upstream factors. Our previous study exposed an evolving mental health crisis in the early 21st century US, which manifested as a nationwide phenomenon when fatal self-injury was represented by SIM rather than suicide, its conventional indicator.^1^ This crisis is intensifying further, given provisional CDC data showing a transformation in the geography of opioid-related fatalities through the westward spread of fentanyl distribution.^32^

Based on our data, which largely preceded the widespread national distribution of fentanyl and its congeners, Western mountain states, adjacent Midwestern states, and Arkansas had the least evidence of misclassifying suicide counts, as they had relatively fewer deaths attributed to fentanyl-related and other drug poisoning deaths than Eastern states prior to 2019. In contrast, states generally east of the Missouri and lower Mississippi rivers bore the greatest burden from the opioid epidemic^1^ and likely the largest adverse impact on their forensic science services.

Exploratory and not explanatory, the LASSO regression analysis revealed potential differential undercounting of SIM. Compared with other states, those with a centralized medical examiner system showed an excess SIM rate adjusting for substantive factors. We hypothesize that centralized medical examiner systems, with their economies of scale and greater investigative resources, were superior to the other systems in distinguishing true-positive drug intoxication deaths among potentially false-positive disease deaths. Affirmation would likely reflect enhanced field investigative capabilities and more autopsy resources, as well as extensive and intensive toxicological testing, advanced imaging procedures, and other sophisticated forensic procedures and practices.^10,33^

Regardless of any apparent impact of the opioid epidemic on states, the type of medicolegal death investigation system was not associated with the reported suicide rate. In contrast to the less rigorous categorization of unintentional and undetermined deaths as the manner of death, the determination of suicide is a strictly defined, conservative process based on a heightened standard of evidence.^14^ Such requirements function to suppress its use and reinforce consistent underreporting across states and regions, irrespective of investigative system type. Medical examiners and coroners have been severely challenged in their work during the opioid epidemic,^19,20,21^ and now the COVID-19 pandemic,^34^ compounded by perennial underresourcing of the forensic sciences.^10^

Contrary to our expectations, our analyses suggested substantial overall differences in the social and demographic determinants we assessed. Two socioeconomic inequity metrics were associated with the SIM rate: positively for the labor underutilization rate and negatively for the percentage of manufacturing employment. Percentage of persons who were uninsured was the only health care access variable to surface as an association in our LASSO analysis, varying negatively with the SIM rate, perhaps spurred by greater access of individuals with health insurance to pharmaceutical opioids, even if subsequently followed by migration to cheaper illicit opioids. Relevant to this possibility, the SIM rate varied positively with the percentage of the adult population prescribed opioids for 30 days or longer.

Two social isolation metrics were associated with the SIM rate: percentage of population that nonreligious was positively associated and the homelessness rate was negatively associated. The second finding is anomalous and calls for comparative research that would include an examination of the medicolegal use of ill-defined and unknown causes in classifying manner of death among individuals experiencing homelessness and consideration of access to treatment, including prescription of pharmaceutical opioids. Firearm ownership was associated with the suicide rate but not the SIM rate, a finding aligning with our expectations, since suicide detection is more self-evident in firearm deaths and SIM focuses intensively on drug fatalities.

While demographic variables yielded 3 expected factors associated with the suicide rate (ie, percentage of population who was male or military veterans, and rural residency status^35,36,37,38^), an unanticipated finding was that neither inequity nor isolation variables were associated. Calling for more research, the latter findings do not imply that there are no associations between their individual-level analogs and the suicide rate. Related to the nature of LASSO, we suggest that while adverse social and economic forces are important contributors to overall population suicide risk, as reported by numerous studies,^25,26,27,30,38^ their impact in our findings was subsumed by demographic variables through our use of an analytic method that seeks to eliminate the effects of collinearity. It was notable that pain reliever misuse was associated with suicide, potentially pointing to the role of chronic disease in suicide and a possible overlap with factors associated with drug-related deaths. This, too, needs further exploration using data beyond the scope of our sources.

Whereas non-Hispanic White race and ethnicity was not associated with the suicide rate, it was associated with the SIM rate, echoing Case and Deaton’s ecological research on so-called *deaths of despair*.^7^ However, SIM, which comprises injury deaths arising from a person’s incident actions, differs fundamentally from this broader construct. Deaths of despair hypothetically encompass all chronic disease deaths associated with an array of health-risk behaviors, such as drinking alcohol (eg, cirrhosis, pancreatitis), smoking (eg, lung cancer, vascular diseases), and poor nutrition (eg, diabetes). In operationalizing their concept, Case and Deaton^7^ omitted these nonalcohol groups and noted excess mortality among less-educated White men and women. We adapted their education variable, percentage of the population aged 25 years and older with a bachelor’s degree, but it was not associated with the SIM rate or the suicide rate. In concert, our findings suggest that SIM is a more representative measure of fatal self-injury than suicide alone.^13^

Societal distress and economic dislocation have been unmistakable sequelae of the COVID-19 pandemic, likely accelerating SIM among persons of color, including Hispanic White, Black, American Indian or Alaska Native, and Asians or Pacific Islander populations, as well as in the majority non-Hispanic White population.^32,39^ Resolving the public health problems posed by harmful behaviors, which induce fatal self-injury, will require implementing proactive, large-scale, diverse, and sustained interventions, embracing community-level social determinants^40^ that affect our economic, legislative, educational, criminal justice, defense, and health care systems. A cultural imperative is the concurrent need to address entrenched stigma across the mental health domain and wider society,^41,42^ and the concomitant tendency to blame the victim.

Among the strengths of this study are its national scope and the application of the enhanced concept of fatal self-injury, SIM. A neglected aspect of research and prevention, this study breaks new ground in incorporating among potential contextual factors associated with interstate SIM and suicide rates the type of medicolegal death investigation system, a proxy for heterogeneity in the quality of investigations. Another strength is our use of LASSO, a statistical procedure that minimizes multicollinearity.

Our work also echoes the call for policy reform from overdose fatality review teams, forensic psychiatrists, and others who strongly recommend quality standards that would apply to medical examiners, coroners, emergency physicians, primary care physicians, and others routinely engaged in the legal and clinical determination of the cause of death.^43^ SIM data are complementary to overdose fatality review and psychological autopsy data, which together may inform local and state health department suicide and overdose prevention strategies.

### Limitations

This study has some limitations. Suicides and drug overdose deaths are local phenomena, even as states are responsible for collecting, coding, and recording mortality data. Our analyses may not have been sufficiently fine-grained: they could not incorporate local variations in some measures, such as inequity, isolation, and rurality. Nonetheless, investigation of state-based, contextual factors could serve as a gateway for informing localized description and analysis. Also, our assessment of data quality was indirect and confined to the system level. A third limitation, likely a conservative one,^6,17^ is that our measurement of the active behavior and nonsuicide drug self-intoxication component of SIM, and thus of SIM itself, was indirect. Direct measurement of SIM could be enabled through the addition of a self-injury checkbox on the death certificate.^44^ A fourth limitation is that there is unexamined heterogeneity in the reliability and validity in some of the substantive contextual variables incorporated in the statistical analysis.

We recognize that LASSO is an atheoretical procedure that is descriptive and exploratory; however, it is a well-suited analytic tool for these initiative investigations. Although we have been intrigued by the potential overlap between those who die by suicide and drug fatalities, our findings underscore different contextual factors may have unique associations with these causes of death.

## Conclusions

The findings of this cross-sectional study suggest that the magnitude of SIM rates was associated with modifiable, upstream factors, including social and environmental. We explored factors associated with both SIM and suicide, given the infeasibility of drawing reliable and valid demarcations between registered suicides and fatalities that reflect instrumentally determined, intentional behaviors, even with no expressed or apparent intention to kill oneself on the day of death.

These results reveal a need to further characterize heterogeneity in medicolegal death investigation processes and data assurance, with the goal of providing the highest quality reports for developing and tracking public health policies and practices. They underscore that while SIM incorporates suicide, with no certain point of demarcation, the overall construct embraces diverse populations, showing fewer overlaps than expected among proposed social determinants. This finding requires thorough investigations using more granular data, additional analytic tools, and focused hypothesis development and evaluation to understand more fully the implications for prevention and intervention.

## Notes

Supplement. **eAppendix.** Testing and Training Datasets for LASSO Regression Analysis
