# Error in Figure 1

**DOI:** 10.1001/jamanetworkopen.2022.2576

**Published:** 2022-03-04

In the Original Investigation titled “Association of State Social and Environmental Factors With Rates of Self-injury Mortality and Suicide in the United States,”^1^ published February 9, 2022, Figure 1 was missing Alaska and Hawaii. This article has been corrected.^1^
