# Supplementary material for: Association of State Social and Environmental Factors With Rates of Self-injury Mortality and Suicide in the United States
Source: JAMA Netw Open. 2022 Feb 9;5(2):e2146591. doi: 10.1001/jamanetworkopen.2021.46591 (PMC8829661; doi:10.1001/jamanetworkopen.2021.46591)
Supplement: Supplement. — eAppendix. Testing and Training Datasets for LASSO Regression Analysis [file jamanetwopen-e2146591-s001.pdf]

Supplemental Online Content

Rockett IRH, Jia H, Ali B, et al. Association of state social and environmental factors with rates of self-injury mortality and suicide in the United States. *JAMA Netw Open*. 2022;5(2):e2146591.
doi:10.1001/jamanetworkopen.2021.46591

eAppendix. Testing and Training Datasets for LASSO Regression Analysis

This supplemental material has been provided by the authors to give readers additional information about their work.

© 2022 Rockett IRH et al. *JAMA Network Open.*

eAppendix. Testing and Training Datasets for LASSO Regression Analysis

Testing dataset: Definitions and data sources for the contextual variables

social inequity/economic resource factors

Median household income is the midpoint that separates the top and bottom halves of the income distribution across households. Data are 1-year estimates for **2017** for the non-institutional population and the source is the *American Community Survey*: https://www.census.gov/content/dam/Census/library/publications/2018/acs/acsbr17-01.pdf

Percentage of population 25 years and older without a bachelor's degree 2017. The source is Table 104.80 reported in the *Digest of Education of Statistics* from the *National Center for Education Statistics*: https://nces.ed.gov/programs/digest/

The **economic dependency ratio** is operationalized as the percentage of the annualized **2017/2018** population aged 0-14 years and 65 years and older divided by the population aged 15-64 years, as indicative of the working age population. The measure is based on population data utilized by *CDC Wide-ranging ONline Data for Epidemiologic Research (WONDER*) in the detailed underlying cause of mortality data: https://wonder.cdc.gov/ucd-icd10.html

The **poverty rate** is the **2017/2018** annualized percentage of people in poverty, derived from the US Census Bureau's *Current Population Surveys*: https://www2.census.gov/programs-surveys/demo/tables/p60/266/state.xls

The **labor underutilization rate** is operationalized as the average of measures for the period from the **third quarter of 2017 through the second quarter of 2018** covering the total unemployed, plus all marginally attached workers *(persons not in the labor force who want and are available for work, and who have looked for a job sometime in the prior 12 months, but were not counted as unemployed because they had not searched for work in the 4 weeks preceding the survey)*, plus total employed part time for economic reasons, as a percentage of the civilian labor force plus all marginally attached workers. The data source was the *US Bureau of Labor Statistics*, *Local Area Unemployment Statistics*: https://www.bls.gov/lau/stalt18q2.htm

The **personal bankruptcy rate is** computed as the number of nonbusiness bankruptcy cases per 1,000 population aged 18 years and older. The data source for the bankruptcies, obtained from the US Courts website, is Table F-2, U.S. Bankruptcy Courts Business and Nonbusiness Cases Commenced, by Chapter of the Bankruptcy Code, During the 12-Month Period Ending December 31, **2018**
https://www.uscourts.gov/sites/default/files/data_tables/bf_f2_1231.2018.pdf

The **housing foreclosure rate** is measured as homes foreclosed per 10,000 housing units, as entered into the *ATTOM Data Solutions* database for September **2017**. *ATTOM Data Solutions* tracks foreclosures: https://www.bankrate.com/finance/real-estate/foreclosures-by-state/default.aspx

Percentage of wage and salary workers in unions in 2018. The data source is Union Membership (Annual) News Release (bls.gov) Table 5. Union affiliation of employed wage and salary workers by state, 2017-2018 annual averages.

Percentage of workforce in manufacturing employment in 2018. The data source is the National Association of Manufacturers State Manufacturing Data | NAM

Social isolation/separation

Calculated as a percentage, a **single-person household**, according to the US Bureau of the Census, refers to households with a single occupant, who is usually a person owning, buying or renting the home in question. The data pertain to **2018** and the data source was *Statista*, which utilized data from the *Current Population Survey*:
https://www.statista.com/statistics/242284/percentage-of-single-person-households-in-the-us-by-state/

Percentage not married was calculated as the combination of the percentage of people 15 years and older in the corresponding total population who were widowed, divorced, never married. The data source is the *US Census Bureau's* ***2018*** *American Community Survey 1-Year Estimates, Table S0101, Age and Sex*:
https://www.census.gov/library/visualizations/interactive/marital-status-in-united-states.html

© 2022 Rockett IRH et al. *JAMA Network Open.*

Percentage of population with residential instability is defined as the percentage of the population aged one year or older who are living in a different residence than in that one year earlier. The data source is the US Census Bureau's ***2018*** *American Community Survey, Table 1. State-to-State Migration Flows1: 2018.* https://www2.census.gov/programs-surveys/demo/tables/geographic-mobility/2018/state-to-state-migration/State_to_State_Migrations_Table_2018.xls

The **homelessness rate** is operationalized as the number of people experiencing homelessness on a single night in January **2018** per 10,000 population. The data source was the *2018 Annual Homeless Assessment Report (AHAR) to Congress PART 1: POINT-IN-TIME ESTIMATES OF HOMELESSNESS DECEMBER 2018*: https://files.hudexchange.info/resources/documents/2018-AHAR-Part-1.pdf

The **incarceration rate** is measured for the 50 states as the number of persons circa 2018 in state prisons, local jails, federal prisons, and other systems of confinement per 100,000 population. With more specifics, the data source, the *Prison Policy Initiative*, is located at: https://www.prisonpolicy.org/global/2018.html

The annualized **homicide rate** for the period **2017/2018** is calculated as deaths due to assault (ICD-10 X85-Y09) and sequelae of assault (Y87.1) divided by the estimated mid-year population and expressed per 100,000 population. As for the outcome measures in this study, the data source is *CDC's Wide-ranging ONline Data for Epidemiologic Research (WONDER)*: Underlying Cause of Death, 1999-2019 Request (cdc.gov)

The **voter turnout rate** is calculated as the percentage of ballots counted for eligible voters (negative prediction). Data were obtained for the **2018** General Election: http://www.electproject.org/2018g

Percentage without broadband service is calculated as the residual of the proportion of households with a broadband internet subscription in 2018. The data source is *Statista*: https://www.statista.com/statistics/185535/us-household-broadband-internet-connection-usage-by-state/

Percentage nonreligious is calculated as the percent of population who reported in **2017** that religion was not important to them or who seldom or never attended religious services. Based on a Gallup Poll, the source is Jim Norman, *The religious regions of the US*, *Politics*, April 6, 2018: https://news.gallup.com/poll/232223/religious-regions.aspx

Demographics

Percentage male is calculated from the state-specific (+ for the District of Columbia) population data from *CDC's Wide-ranging ONline Data for Epidemiologic Research (WONDER)* for the period **2017/2018**: Underlying Cause of Death, 1999-2019 Request (cdc.gov)

Percentage non-Hispanic white is calculated from the state-specific (+ for the District of Columbia) population data from *CDC's Wide-ranging ONline Data for Epidemiologic Research (WONDER)* for the period **2017/2018**: Underlying Cause of Death, 1999-2019 Request (cdc.gov)

Percentage civilian military veterans 2018 measured as the percentage of military veterans in the adults in the civilian population aged 18 years and older. The data source is the 2018 American Community Survey 1-year estimates. Table S2101 Veteran Status: https://data.census.gov/cedsci/table?text=Table%20S2101%20Veteran%20Status&tid=ACSST1Y2018.S2101&hidePreview=true

Percentage rural population is determined by the **2010** US census population enumeration. The data source is the US Census Bureau: https://data.census.gov/cedsci/table?q=rural%20urban&g=0100000US.04000.001&tid=DECENNIALSF12010.H2&hidePreview=true&tp=true

Geographic regions 4 major (West/Midwest/Northeast/South) accord with the Divisions used in the **2010** US census. The data source is the US Census Bureau: https://www.census.gov/geographies/reference-maps/2010/geo/2010-census-regions-and-divisions-of-the-united-states.html

© 2022 Rockett IRH et al. *JAMA Network Open.*

Injury mechanism

Alcohol consumption in 2016 is measured per capita as apparent consumption of ethanol in gallons, based on the population ages 14 years and older. The data source was Haughwout SP, Slater ME (2018) *Apparent Per Capita Alcohol Consumption: National, State, and Regional Trends, 1977-2016.* U.S. Department of Health and Human Services, National Institutes of Health, Bethesda, MD: https://pubs.niaaa.nih.gov/publications/surveillance110/tab2_16.htm

Percentage firearm ownership rate, 2013. The data source is Kaleson B, Villarreal MD, Keyes KM, and Galea S. "Gun ownership and social gun culture." *Injury Prevention* 2015; 22:3. https://injuryprevention.bmj.com/content/injuryprev/early/2015/06/09/injuryprev-2015-041586.full.pdf?keytype=ref&ijkey=doj6vx0laFZMsQ2

Percentage of opioids prescribed in the population for 30 days or more measured for **2017**. The data source is eTable 4 in Schieber LZ, Guy GP, Seth P, et al. Trends and patterns of geographic variation in opioid prescribing practices by state, United States, 2006-2017. *Jama Network Open*, 2019; 2(3):e190665; https://jamanetwork.com/journals/jamanetworkopen/fullarticle/2728005

Prevalence of illicit drug use other than marijuana in the past month measured as the **2017/2018** annual average of the percentage of survey respondents aged 18 years and older. The data source is Table 6 from the *2017-2018 National Survey on Drug Use and Health: Model-Based Prevalence Estimates (50 States and the District of Columbia)*: https://www.samhsa.gov/data/sites/default/files/reports/rpt23235/2k18SAEExcelTabs/NSDUHsaePercents2018.pdf

Prevalence of pain reliever misuse in the past year measured as the **2017/2018** annual average of the percentage of survey respondents aged 18 years and older. The data source is Table 12 from the *2017-2018 National Survey on Drug Use and Health: Model-Based Prevalence Estimates (50 States and the District of Columbia)*: https://www.samhsa.gov/data/sites/default/files/reports/rpt23235/2k18SAEExcelTabs/NSDUHsaePercents2018.pdf

Healthcare access

Percentage of adults without a usual place of medical care. Adults are defined as individuals ages 18–64 years. Data are based on findings from the Centers for Disease Control and Prevention (CDC), National Center for Health Statistics (NCHS) National Health Interview Survey (NHIS), **2014**. The data source is Black LI, Schiller JS. [State variation in health care service utilization: United States, 2014]. *NCHS data brief*, no 245. Hyattsville, MD: National Center for Health Statistics. May 2016. https://www.kff.org/other/state-indicator/percent-of-adults-without-a-usual-place-of-medical-care/?currentTimeframe=0&sortModel=%7B%22colId%22:%22Location%22,%22sort%22:%22asc%22%7D

Percentage of adults reporting unmet need for mental health treatment is measured as the percentage of adult respondents ages 18 years or older who felt a perceived need for mental health treatment/counseling in the past year that was not received, often referred to as "unmet need." The data source is the Kaiser Family Foundation analysis of Substance Abuse and Mental Health Services Administration (SAMHSA)'s restricted online data analysis system (RDAS), *National Survey on Drug Use and Health* (NSDUH), **2017 and 2018**, Substance Abuse and Mental Health Data Archive. https://www.kff.org/other/state-indicator/adults-reporting-unmet-need-for-mental-health-treatment-in-the-past-year/?currentTimeframe=0&sortModel=%7B%22colId%22:%22Location%22,%22sort%22:%22asc%22%7D

Percentage without health insurance is measured as the percentage of the civilian noninstitutionalized population of the US in **2017** lacking health insurance coverage. The data source is Table 6 in a US Bureau of the Census report entitled *Health Insurance Coverage in the United States: 2017*: https://www2.census.gov/programs-surveys/demo/tables/p60/264/table6.pdf

Percentage of adults with unfavorable disability claim ruling 2018 (Fiscal Year) is measured as 100 minus the percentage of adults with favorable rulings from a state agency. The data source is the Social Security Administration, *SSA Fiscal Year Disability Claim Data*. https://www.ssa.gov/disability/data/SSA-SA-FYWL.csv

Type of medicolegal death investigation system

Death Investigation systems by state are distinguished as **centralized state medical examiner, decentralized county/district medical examiner, medical examiner and coroner hybrid, and decentralized county coroner**. Source: https://www.cdc.gov/phlp/publications/coroner/death.html plus consultation with Dr. Kurt B. Nolte, former Chief Medical Examiner for New Mexico and past Executive Vice-President of the National Association of Medical Examiners.

© 2022 Rockett IRH et al. *JAMA Network Open.*

Training dataset: Definitions and data sources for the contextual variables

social inequity/economic resource factors

Median household income is the midpoint that separates the top and bottom halves of the income distribution across households. *Table 1. Median Household Income and Gini Index in the Past 12 Months by State and Puerto Rico: 2015 and **2016***. Household Income: 2016 (census.gov)

Percentage of population 25 years and older without a bachelor's degree 2016. The source was *Table 104.80 reported in the Digest of Education of Statistics from the National Center for Education Statistics 2017*: https://nces.ed.gov/programs/digest/

The **economic dependency ratio** was operationalized as the percentage of the annualized **2016/2017** population aged 0-14 years and 65 years and older divided by the population aged 15-64 years, as indicative of the working age population. The measure is based on population data utilized by CDC *Wide-ranging ONline Data for Epidemiologic Research* (WONDER) in the detailed underlying cause of mortality data: https://wonder.cdc.gov/ucd-icd10.html

The **poverty rate** was the **2015/2016** annualized percentage of people in poverty, derived from the US Census Bureau's *Current Population Surveys, 2014 to 2017*: https://www.census.gov/library/publications/2017/demo/p60-259.html

The **labor underutilization** rate was operationalized as the average of measures for the period from the **third quarter of 2016 through the second quarter of 2017** covering the total unemployed, plus all marginally attached workers (persons not in the labor force who want and are available for work, and who have looked for a job sometime in the prior 12 months, but were not counted as unemployed because they had not searched for work in the 4 weeks preceding the survey), plus total employed part time for economic reasons, as a percentage of the civilian labor force plus all marginally attached workers. The data source was the US Bureau of Labor Statistics, *Local Area Unemployment Statistics*: https://www.bls.gov/lau/stalt17q2.htm

The **personal bankruptcy rate** was computed as the number of nonbusiness bankruptcy cases per 1,000 population aged 18 years and older. The data source for the bankruptcies, obtained from the US Courts website, was *Table F-2, U.S. Bankruptcy Courts Business and Nonbusiness Cases Commenced*, by Chapter of the Bankruptcy Code, During the 12-Month Period Ending December 31, **2016**. https://www.uscourts.gov/sites/default/files/data_tables/stfj_f2_1231.2016.pdf

The **housing foreclosure rate** was measured as homes foreclosed per 10,000 housing units, as entered into the ATTOM Data Solutions database for September **2017**. *ATTOM Data Solutions tracks foreclosures*: https://www.bankrate.com/finance/real-estate/foreclosures-by-state/default.aspx

Percentage of wage and salary workers in unions in 2016. The data source is Table 5. Union affiliation of employed wage and salary workers by state, 2015-2016. UNION MEMBERS-2016 (bls.gov)

Percentage of workforce in manufacturing employment in 2016. Percentage of workforce in manufacturing employment (in 2016). The data source was the National Association of District Export Councils: https://www.usaexporter.org/ Data were missing for Illinois and the District of Columbia (DC). In the case of Illinois an estimate from a Nebraska report, based on preliminary interstate manufacturing data, was used: https://nemep.unl.edu/downloads/1605.052_NMEP-Large-Report.pdf. For DC, 2018 data were used. Source: the National Association of Manufacturers https://nemep.unl.edu/downloads/1605.052_NMEP-Large-Report.pdf

Social isolation/separation

Calculated as a percentage, a **single-person household**, according to the US Bureau of the Census, refers to households with a single occupant, who is usually a person owning, buying or renting the home in question. The data source was the US Census Bureau's ***2016** American Community Survey* 1-Year Estimates, Table S1101, Households and Families: https://data.census.gov/cedsci/table?q=singe%20person%20household%202016&text=S1101&g=0100000US.04000.001&tid=ACSST1Y2016.S1101&hidePreview=true

Percentage of population with residential instability is defined as the percentage of the population aged one year or older who are living in a different residence than in that one year earlier. Data source was the U.S. Census Bureau's **2016** *American Community Survey*, Table 1. State-to-State Migration Flows: 2016**.** https://www2.census.gov/programs-surveys/demo/tables/geographic-mobility/2016/state-to-state-migration/State_to_State_Migrations_Table_2016.xls

© 2022 Rockett IRH et al. *JAMA Network Open.*

The **homelessness rate** was operationalized as the percentage of people experiencing homelessness on a single night in January **2016**. The data source was *The 2016 Annual Homeless Assessment Report (AHAR) to Congress PART 2: Estimates of Homelessness in the United States, December 2017*. EXHIBIT 2.3: Homeless Individuals in the U.S. Percentage of National Total in Each State, 2016: The 2016 Annual Homeless Assessment Report (AHAR) to Congress, Part 2: Estimates of Homelessness in the U.S. (huduser.gov)

The **incarceration rate** was measured for the 50 states as the number of persons circa **2016** in state prisons, local jails, federal prisons, and other systems of confinement per 100,000 population. With more specifics, the data source, the *Prison Policy Initiative*, is located at: States of Incarceration: The Global Context 2016 | Prison Policy Initiative

The annualized **homicide rate** for the period **2016/2017** was calculated as deaths due to assault (ICD-10 X85-Y09) and sequelae of assault (Y87.1) divided by the estimated mid-year population and expressed per 100,000 population. As for the outcome measures in this study, the data source was CDC's *Wide-ranging ONline Data for Epidemiologic Research* (WONDER): Underlying Cause of Death, 1999-2019 Request (cdc.gov)

The **voter turnout rate** was calculated as the percentage of ballots counted for eligible voters (negative prediction). Data were obtained for the **2016** *General Election*: 2016g - United States Elections Project (electproject.org)

Percentage without broadband service was calculated as the residual of the proportion of households with a broadband internet subscription in **2016**. The data source was Computer and Internet Use in the United States: *2016, American Community Survey Reports*. Table 5. Percentage of Households with a Broadband Internet Subscription: 2016: Computer and Internet Use in the United States: 2016 (census.gov)

Percentage nonreligious was calculated as the percent of population who reported in **2017** that religion was not important to them or who seldom or never attended religious services. Based on a Gallup Poll, the source was Jim Norman, *The religious regions of the US, Politics, April 6, 2018*: https://news.gallup.com/poll/232223/religious-regions.aspx

Demographics

Percentage male was calculated from the state-specific (+ for the District of Columbia) population data from CDC's *Wide-ranging ONline Data for Epidemiologic Research* (WONDER) for the period **2016/2017**: Underlying Cause of Death, 1999-2019 Request (cdc.gov)

Percentage non-Hispanic white was calculated from the state-specific (+ for the District of Columbia) population data from CDC's *Wide-ranging ONline Data for Epidemiologic Research* (WONDER) for the period **2016/2017**: Underlying Cause of Death, 1999-2019 Request (cdc.gov)

Percentage civilian military veterans 2016 measured as the percentage of military veterans in the adults in the civilian population aged 18 years and older. The data source was the US Census Bureau's *2016 American Community Survey* 1-Year Estimates, Table S2101, Veteran Status: https://data.census.gov/cedsci/table?q=ACSST1Y2019.S2101&g=0100000US.04000.001&tid=ACSST1Y2016.S2101&hidePreview=true&tp=true

Percentage rural population was determined by the **2010** US census population enumeration. The data source was the *US Census Bureau*: https://data.census.gov/cedsci/table?q=rural%20urban&g=0100000US.04000.001&tid=DECENNIALSF12010.H2&hidePreview=true&tp=true

Geographic regions 4 major (West/Midwest/Northeast/South) accorded with the Divisions used in the **2010** US census. The data source was the *US Census Bureau*: https://www.census.gov/geographies/reference-maps/2010/geo/2010-census-regions-and-divisions-of-the-united-states.html

Injury mechanism

Alcohol consumption in 2016 was measured per capita as apparent consumption of ethanol in gallons, based on the population ages 14 years and older. The data source was Haughwout SP, Slater ME (2018) *Apparent Per Capita Alcohol Consumption: National, State, and Regional Trends, 1977-2016*. U.S. Department of Health and Human Services, National Institutes of Health, Bethesda, MD: https://pubs.niaaa.nih.gov/publications/surveillance110/tab2_16.htm

© 2022 Rockett IRH et al. *JAMA Network Open.*

Percentage firearm ownership rate, 2013. The data source was Kaleson B, Villarreal MD, Keyes KM, and Galea S. *"Gun ownership and social gun culture." Injury Prevention 2015*; 22:3. https://injuryprevention.bmj.com/content/injuryprev/early/2015/06/09/injuryprev-2015-041586.full.pdf?keytype=ref&ijkey=doj6vx0laFZMsQ2

Percentage of opioids prescribed in the population for 30 days or more measured for **2017**. The data source was eTable 4 in Schieber LZ, Guy GP, Seth P, et al. *Trends and patterns of geographic variation in opioid prescribing practices by state, United States, 2006-2017. Jama Network Open, 2019*; 2(3):e190665; https://jamanetwork.com/journals/jamanetworkopen/fullarticle/2728005

Prevalence of illicit drug use other than marijuana in the past month measured as the **2015/2016** annual average of the percentage of survey respondents aged 18 years and older. The data source was *Table 6 from the 2015-2016 National Survey on Drug Use and Health*: Model-Based Prevalence Estimates (50 States and the District of Columbia): https://www.samhsa.gov/data/sites/default/files/NSDUHsaePercents2016/NSDUHsaePercents2016.pdf

Prevalence of pain reliever misuse in the past year measured as the **2015/2016** annual average of the percentage of survey respondents aged 18 years and older. The data source was *Table 11 from the 2015-2016 National Survey on Drug Use and Health*: Model-Based Prevalence Estimates (50 States and the District of Columbia): https://www.samhsa.gov/data/sites/default/files/NSDUHsaePercents2016/NSDUHsaePercents2016.pdf

Healthcare access

Percentage of adults without a usual place of medical care. Adults were defined as individuals ages 18–64 years. Data were based on findings from the Centers for Disease Control and Prevention (CDC), National Center for Health Statistics (NCHS) National Health Interview Survey (NHIS), **2014**. The data source was Black LI, Schiller JS. *State variation in health care service utilization: United States, 2014. NCHS data brief*, no 245. Hyattsville, MD: National Center for Health Statistics. May 2016. https://www.kff.org/other/state-indicator/percent-of-adults-without-a-usual-place-of-medical-care/?currentTimeframe=0&sortModel=%7B%22colId%22:%22Location%22,%22sort%22:%22asc%22%7D

Percentage of adults reporting unmet need for mental health treatment was measured as the percentage of adult respondents with any mental illness who reported unmet treatment need. The data source was Mental Health America's report entitled *Prevention and Early Intervention B4Stage4: The State of Mental Health in America* **2016**. https://www.mhanational.org/sites/default/files/2019-07/2016%20MH%20in%20America%20FINAL.pdf

Percentage without health insurance was measured as the percentage of the civilian noninstitutionalized population of the US in **2016** lacking health insurance coverage. The data source was Table 6 in a US Bureau of the Census report entitled *Health Insurance Coverage in the United States: 2016*: Income, Poverty, and Health Insurance Coverage in the United States: 2010 (census.gov)

Percentage of adults with unfavorable disability claim ruling 2016 (Fiscal Year) was measured as 100 minus the percentage of adults with favorable rulings from a state agency. The data source was the *Social Security Administration, SSA Fiscal Year Disability Claim Data*. https://www.ssa.gov/disability/data/SSA-SA-FYWL.csv

Type of medicolegal death investigation system

Death Investigation systems by state were distinguished as **centralized state medical examiner, decentralized county/district medical examiner, medical examiner and coroner hybrid, and decentralized county coroner**. Source: M. Warner et al. State Variation in Certifying Manner of Death and Drugs Involved in Drug Intoxication Deaths. *Acad Forensic Pathol. 2013* 3 (2): 231-237, plus consultation with Dr. Kurt B. Nolte, former Chief Medical Examiner for New Mexico and past Executive Vice-President of the National Association of Medical Examiners.

© 2022 Rockett IRH et al. *JAMA Network Open.*
